# Traditional Ceremonial Practices as a Strategy to Reduce Problem Substance Use in American Indian Communities: A Systematic Review

**DOI:** 10.1089/jicm.2022.0655

**Published:** 2023-06-06

**Authors:** Damian M. Chase-Begay, Jeffery Chaichana Peterson, Jessica Liddell, Annie Belcourt

**Affiliations:** ^1^ School of Public and Community Health Sciences, University of Montana, Missoula, MT, USA.; ^2^ School of Social Work, University of Montana, Missoula, MT, USA.

**Keywords:** traditional practices, ceremony, American Indian, Native American, substance use, systematic review

## Abstract

**Objective:**

This systematic review assessed the feasibility of American Indian traditional ceremonial practices (TCPs) to address problem substance use in both reservation and urban settings.

**Methods:**

Between September 24, 2021, and January 14, 2022, culturally specific review protocols were applied to articles retrieved from over 160 electronic databases—including PubMed, Global Health, Global Health Archive, CINAHL Complete, PsychInfo, Web of Science, Health and Wellness (Gale), Sage Online Journals, and ScienceDirect.

**Results:**

A total of 10 studies met the criteria for inclusion in the review. Studies were conducted with both urban (*n* = 7) and reservation (*n* = 3) American Indian and Alaska Native (AIAN) populations. The most common TCP activities reported were drumming (*n* = 9), sweat lodge (*n* = 7), and talking circles (*n* = 6). All 10 studies reported some type of quantitative data showing a reduction of substance use associated with TCP interventions or activities.

**Conclusions:**

The current status of the literature is emerging and does not allow for meta-analysis of existing studies. However, the existing literature does indicate promise for the use of TCPs to address problem substance use in AIAN communities in a way that is effective and also culturally congruent.

## Introduction

American Indian and Alaska Native (AIAN)* communities have successfully relied on long-held traditional ceremonial practices (TCPs) to survive and recover from historical traumas for generations.^1^ Interventions that incorporate TCPs to prevent or treat problem substance use are increasingly replacing the more deficit-based clinical approaches employed by Western science.^2–4^ Beyond merely introducing or strengthening aspects of culture—such as language, foods, games, or arts and crafts—AIAN communities are reviving TCPs in an effort to promote spiritual and emotional healing and, correspondingly, reduce rates of problem substance use associated with historical and intergenerational trauma.^5^

In its 2018 *Declaration of Astana*,^6^ the Global Conference on Primary Health Care acknowledged the growing burden that problem substance use places on marginalized and underserved communities. As part of this declaration, leaders from nations around the world committed to empowering individuals and communities to actively participate in informing and implementing policies and practices that impact on their health. They also committed to supporting the development of policies and practices that incorporate traditional knowledge and wisdom.

Increased efforts to incorporate TCPs into substance use programming are, in many ways, a form of contemporary resistance for many Indigenous peoples. Historically, it is important to remember that many of these practices were undervalued, disregarded, and even deemed illegal in the United States, and this has resulted in a persistent distrust of research on the part of AIAN communities.^7,8^ These factors have helped fuel a relative paucity of Western scientific evidence regarding the effectiveness of TCPs. This presents a challenge for administrators of substance use programs, because it has led to a current policy and funding environment in which, despite millennia of practice-based evidence supporting their effectiveness, traditional practitioners and TCP-based interventions are often deemed ineligible for reimbursement through public and private insurers.^9^

This trend is beginning to change, however, as greater emphasis is placed on community-based participatory research methods and as more research is conducted *by* AIAN investigators *for* AIAN communities.^3^ Studies have emerged over the last few decades, and are increasing in number, which specifically explore the effectiveness of TCPs to reduce problem substance use. One issue, however, is that many of these studies take place outside the United States and focus on Indigenous communities in countries such as Canada, Australia, New Zealand, or other parts of the world.^10–12^ For studies inside the United States, AIAN adolescents are often the focus, due, in part, to the severity of health disparities in substance use-related mortality for AIAN teens.^13–15^ How successfully these extraterritorial and youth-focused studies might generalize to AIAN adults is not well established.

Another issue is that Indigenous and community-based participatory research methods can seem to lend themselves more easily to a narrative, or qualitative, inquiry. This is perhaps due to the emphasis on relational communal values, rather than a more compartmentalized, quantitative approach to understanding health.^16^ This effect has resulted in a greater preponderance of *qualitative* literature exploring the role and effectiveness of TCP in reducing problem substance use in AIAN communities.^8,17,18^ This is problematic because quantitative methods are often prioritized by funding institutions and scientists, even though qualitative methods are an important source of insight for examining indigenous healing methods.

The gap within the extant literature of studies that examine cross-cultural health and healing with the inferential power of quantitative methods adds to the continued designation of TCPs as a “nonevidence-based” practice. This project seeks to fill this lacuna by providing a review of *quantitative* data to address the question: What are the associations between interventions that incorporate TCPs and problem substance use, and do the relationships vary by reservation or urban setting?

### What are TCPs?

Given the significant heterogeneity among tribal nations throughout the United States, activities that are considered TCPs are diverse. Most tribes consider TCPs as ethically protected tribal knowledge. However, some examples of TCPs include sweat lodge, drumming, talking circles, naming ceremonies, puberty ceremonies, or other spiritual ceremonies practiced by tribal nations.

This review was guided by Critical Indigenous Research Methodologies (CIRM).^19^ A basic tenet of CIRM is tribal self-determination. This means that Indigenous communities have the right to identify an activity as a TCP, without a need to define or operationalize the practice for researchers. Thus, for the purposes of this review, the research team considered an activity or intervention as a TCP if that is how it was regarded by the respective AIAN communities included in the studies. The intent of this review is to potentially inform the development and delivery of TCP-based interventions with AIAN adults in the United States.

## Methods

The study team consisted of four researchers, two of whom are American Indian and two of whom are not, but who have extensive backgrounds working with AIAN populations. The researchers come from four distinct social science fields—public health, health communications, social work, and clinical psychology—to enhance the perspective of the team when reviewing studies from various disciplines. On September 24, 2021, a systematic search of the literature was conducted in accordance with the Preferred Reporting Items for Systematic Reviews and Meta-Analyses (PRISMA) statement.^20,21^ The full review protocol was registered with PROSPERO (ID No. CRD42021269710) before the search was conducted and can be found in Supplementary Appendix SA1.

### Search terms

Because the introduction of TCPs into clinical and academic study is relatively recent, there is no standardized language to facilitate literature reviews. To ensure as many relevant studies as possible were captured, a broad search strategy was employed. The search terms were informed by meetings with behavioral health staff from several Urban Indian Health Organizations (UIHOs). These staff identified the substances most often associated with problem use by their AIAN clientele as alcohol, methamphetamines, and opioids. Because of this, the search included the terms “substance use,” “substance abuse,” and “addiction,” as well as terms for the three substances specifically singled out by UIHO staff (Table 1).

**Table 1.** Search Terms for the PRISMA-Guided Systematic Review

American Indian (OR) Native American (OR) Indigenous	(AND)	Spiritual^*^ (OR) Ceremon^*^ (OR) Cultur^*^ (OR) Tradition^*^ (OR) Community	(AND)	Substance Use (OR) Substance Abuse (OR) Addiction (OR) Alcohol Use (OR) Alcohol Abuse (OR) Meth^*^ Use (OR) Meth^*^ Abuse (OR) Opioid Use (OR) Opioid Abuse (OR) Prescription Medicine (OR) Prescription Drug

PRISMA, Preferred Reporting Items for Systematic Reviews and Meta-Analyses.

### Article selection

Through a university library OneSearch function, >160 electronic databases were searched, including PubMed, Global Health, Global Health Archive, CINAHL Complete, PsychInfo, Web of Science, Health and Wellness (Gale), Sage Online Journals, and ScienceDirect. References were uploaded to EndNote (version 20.2) for organization and review.

A limited gray literature search was also performed. After entering the search terms into both Google Search and Google Scholar, websites for community-based organizations, such as National Congress of American Indians, National Council for Urban Indian Health, and Urban Indian Health Institute, and government agencies such as the Indian Health Service (IHS), Substance Abuse and Mental Health Services Administration, and National Institutes of Health, were reviewed in search of published data. Studies and projects located on these websites had either already been identified through databases or were not eligible for inclusion. Therefore, the research team decided to focus its review on the academic literature.

The first and second authors (D.B. and J.C.P., respectively) independently screened each title and then each remaining abstract for inclusion. Abstracts were screened against a guideline document^22^ to determine whether full article review was appropriate (see abstract review guideline document in Supplementary Appendix SA2). D.B. and J.C.P. resolved discrepancies through discussion until consensus was reached. Full articles were then initially reviewed against a guideline by D.B. and were assigned as exclude, potentially exclude, or potentially include. Those articles that were assigned as potentially exclude or potentially include were then brought to the full research team for discussion and final consensus.

### Study inclusion/exclusion

The original intent of the review was to include studies that specifically tested interventions. Due to the low number of these types of studies, cross-sectional studies in which both a TCP and substance use measure were clearly defined and reported on were also included. Because this field of research is not widely standardized, the research team did not expect to find enough consistency among how studies measured effect, for example, odds of abstaining or the number of alcoholic drinks/times a substance was consumed during a specific period of time, to conduct a meta-analysis.

Search parameters included all articles published in English, with full text available, and no date restriction was applied. Studies were included if they (1) explored the association between TCP and substance use prevention or treatment for AIAN adults using a quantitative design and (2) met the criteria for addressing cultural adherence as outlined below.

### Study bias

Risk of bias (RoB) among the studies was measured using several different instruments. The single randomized control trial was assessed using RoB 2, the updated recommended Cochrane tool.^23^ This tool provides a framework for evaluating the potential risk for bias from any type of randomized trial. Each randomized clinical trial (RTC) is judged on five domains, with each domain categorized as high RoB, low RoB, or some concerns. The prospective cohort and intervention studies were assessed for quality using ROBINS-I, the recommended Cochrane tool.^24^

Similar to the RoB 2, this tool evaluates the potential risk for bias over six domains, with each domain categorized as having a low, moderate, serious, or critical RoB. Finally, the cross-sectional studies were assessed for quality using the NIH Quality Assessment Tool for Observational Cohort and Cross-Sectional Studies.^25^ This list contains 14 items evaluating quality criteria, and quality scores are assigned based on the number of criteria addressed adequately in the study.

### Cultural adherence

As mentioned in the introduction, there exists a historical gap between Indigenous wisdom and Western science. Significant advancement has been made to narrow this gap, including the establishment in 2000 of the Native American Research Centers for Health (NARCH). This program is a partnership between the National Institutes of Health (NIH) and the IHS.^26^ By 2018, however, NARCH funding represented less than one-half of one percent of the NIH budget.^27^

The chronic underfunding of AIAN health research, and the persistent distrust many AIAN communities have developed after experiencing exploitation by researchers, must be considered when examining the quality of studies undertaken with this population. Because of this, the research team decided to assess cultural, in addition to methodological, adherence among the studies. Before including an article in this review, it was examined to determine (1) whether the research was identified as either Indigenous led or Indigenous informed, (2) that TCP design was overseen or directly delivered by AIAN traditional practitioners, and (3) that implementation and evaluation processes were culturally justified in the narrative.

For example, a study examining the effect of sweat lodge ceremonies on problem substance use in the Navajo Nation included a traditional counselor as its second author, employees of both the Navajo Nation and the IHS as additional authors, and the narrative included information as to how the intervention and evaluation methods were grounded in Navajo tradition and culture.^28^ The review team argues that by meeting these criteria, this and other studies contribute significantly to an area of research that could benefit greatly from future increases in attention and resources.

However, this conversely led to a situation in which some articles were excluded, even though they might potentially add benefit to this conversation. For example, two studies by Venner et al.^29,30^ examined a culturally adapted Motivational Interviewing and Community Reinforcement Approach. The narrative of one study noted that, “Referral to one of the three cultural educators was available to provide Tribal traditional teachings.”^29(p.953)^ Nevertheless, there was no information about whether these teachings included TCPs or how those TCPs were either overseen in design or directly delivered by traditional practitioners. The research team recognizes that most tribes consider TCPs to be ethically protected tribal knowledge and there are many valid reasons why researchers might not explicate in great detail on practices and protocols. The process applied in this review was an earnest attempt to acknowledge tribal sovereignty, while also assessing TCP legitimacy to the greatest extent possible.

## Results

**FIG. 1.**
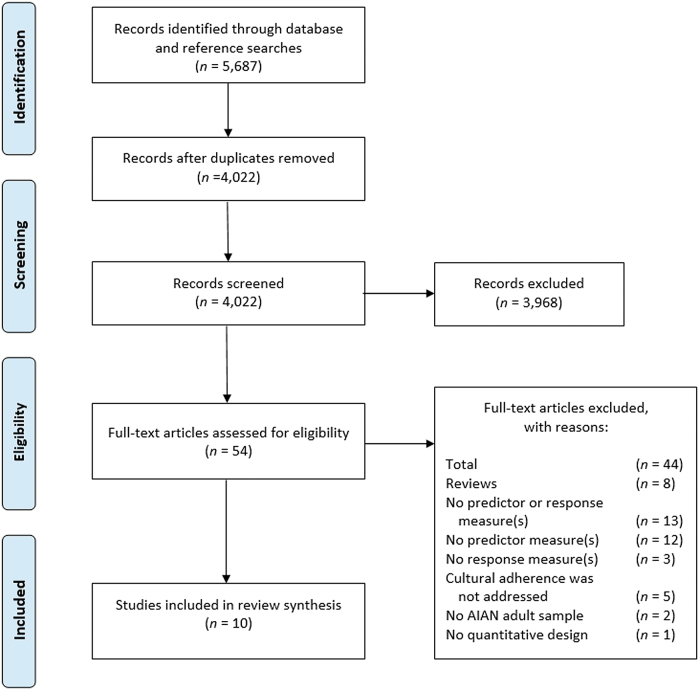
PRISMA Flow Diagram for the systematic review.

Figure 1 provides a detailed outline of the article selection process. After screening, 54 full text articles were reviewed to see if they met the inclusion criteria. Of these, 44 articles were excluded for the following reasons: (1) other reviews, including systematic reviews, scoping reviews, and meta-analyses (*n* = 8); (2) no predictor (TCP) or response (substance use) measure was reported (*n* = 13); (3) no predictor measure (TCP) was reported (*n* = 12); (4) no response measure (substance use) was reported (*n* = 3); (5) cultural adherence was not addressed (*n* = 5); (6) the study did not include an AIAN adult sample (*n* = 2); and (7) the study did not employ a quantitative design (*n* = 1).

A total of 10 studies reporting quantitative measures on AIAN adults met the inclusion criteria for the review. They were as follows: one randomized control trial; one pilot study; one prospective cohort study; four evaluation studies; and three cross-sectional studies (Table 2). All 10 studies were published in academic journals between 2003 and 2021. Study environments included both reservations (*n* = 3) and urban settings (*n* = 7). Five of the studies targeted participants in either an inpatient or outpatient substance use treatment program, two studies targeted specific reservation communities, one study targeted inmates, one study targeted homeless individuals, and one study targeted college students.

**Table 2.** Summary of the Characteristics and Results of the Included Studies (*N* = 10)

Study	Design	Sample size urban/reservation	Predictor/exposure	Response/outcome	Results
1. Dickerson et al. (2021)^32^	Randomized clinical trial	*N* = 63 Urban	Twelve 3-h sessions (total of 36 h) of therapeutic drum behavior therapy for AI/AN adults with AOD use disorders.	Quantity and frequency of substance use by participants.	DARTNA participants reported fewer drinks per day (*d* = 0.39, 95% CI = −1.04 to 0.27), and lower odds of marijuana use in past 30 days (odds ratio = 0.50, 95% CI = 0.10–2.54) compared to usual care.
2. Dickerson et al. (2014)^31^	Pilot study	*N* = 10 Urban	3-Hour treatment of therapeutic drum behavior therapy protocol sessions provided two times per week for 12 weeks.	Quantity and frequency of substance use by participants.	75% (six of eight participants) reported no alcohol or drug use at follow-up.
3. Gossage et al. (2003)^28^	Evaluation study	*N* = 190 Reservation	Navajo traditional practitioners conducted sweat lodge ceremonies with I/Ps.	Quantity and frequency of substance use by participants.	At follow-up, inmate/patients (I/Ps) were drinking about 1.5 drinks less than before the intake data were collected (5.4 vs. 6.8).
4. Greenfield et al. (2018)^39^	Cross-sectional survey	*N* = 347 Urban	Survey scale for *Traditional Spiritual Activities.* Response options on a 4-point Likert rating scale.	Quantity and frequency of substance use by participants.	Lower proportion of past-month drug use among respondents who participate in traditional ceremony: 16.1% and 25.5%, respectively (*p* = 0.041). Lower proportion of past-month marijuana use among respondents who participate in traditional ceremony: 10.5% and 17.4%, respectively (*p* = 0.079). Lower proportion of past-month alcohol use among respondents who participate in traditional ceremony: 39.3% and 48.3%, respectively (*p* = 0.102). Lower proportion of past-month binge drinking among respondents who participate in traditional ceremony: 23.6% and 30.5%, respectively (*p* = 0.177).
5. Herman-Stahl et al. (2003)^33^	Cross-sectional survey	*N* = 2449 Reservation	Survey items asking about participation in traditional practices.	Quantity and frequency of substance use by participants.	“More American Indian oriented” were less likely to report alcohol use than “bicultural” and “less American Indian oriented” (20.4%, 51.1%, and 58.6%, respectively; *p* = <0.001); less likely to report heavy alcohol use (11.2%, 28.8%, and 39.9%, respectively; *p* = <0.001); and less likely to report illicit drug use (12.2%, 26.9%, and 27.9%, respectively; *p* = <0.001). Compared to “more American Indian oriented” heavy drinking OR for “bicultural” was 2.88 [95% CI (1.85–4.47); *p* = <0.05] and for “less American Indian oriented” was 4.38 [95% CI (2.56–7.49); *p* = <0.05].
6. Saylors (2003)^34^	Evaluation study	*N* = 742 Urban	AIAN cultural healing activities, including prayer, singing, drumming, sweat lodges, smudging, herbs, and use of tobacco in ceremonies. Talking circles were held regularly at the clinic for clients.	Quantity and frequency of substance use by participants.	Within the prematched/postmatched sample, alcohol use decreased 13% after 6 months and drinking alcohol to intoxication was reduced by 19%. Of those women who had used marijuana, nonprescription methadone, hallucinogens, uppers, downers, and inhalants at intake, none reported use at 6 months. Heroin use was down 93%.
7. Tonigan et al. (2020)^38^	Prospective cohort	*N* = 61 Urban	Frequency of attendance at either standard AA or AA that was CA-AA to include TCP.	Two alcohol use measures were computed: proportion of days abstinent from alcohol and drinks per drinking day	CA-AA participants reported an average of 6.49 drinks per drinking day compared to 6.72 for the AA-only participants.
8. Torres-Stone et al. (2006)^36^	Cross-sectional survey	*N* = 980 Reservation	Survey scale for T*raditional Spirituality Scale.* Response options on a 4-point Likert rating scale.	Alcohol cessation was measured as a dichotomous variable, indicating they no longer drank alcohol after a prior period of alcohol use or abuse.	Alcohol cessation was significantly and positively associated with participation in traditional spiritual activities (*r* = 0.23, *p* = <0.01).
9. Wendt et al. (2017)^37^	Evaluation study	*N* = 52 (AIAN subsample) Urban	Native-specific TCP activities were coded from participants' brief, open-ended responses by a Native coauthor.	Quantity and frequency of substance use by participants.	AIANs who engaged in TCP reported significantly lower drinking frequency in past 30 days (mean = 10.00 days vs. 24.15 days, *p* = 0.009), as well as amount consumed in the last 30 days (mean = 10.34 vs. 31.25, *p* = 0.017). Marginally significant difference in days of intoxication, with fewer days of intoxication among those who engaged in TCP (Mdn = 5.00 vs. Mdn = 29.50, *p* = 0.05).
10. Wright et al. (2011)^35^	Evaluation study	*N* = 490 Urban	Integrated mental health and substance abuse system that includes traditional cultural practices: talking circles, sweat lodge, traditional healers, seasonal ceremonies, prayer, smudging, drumming, and herbs.	Frequency of substance use by participants.	An 80.2% decrease in alcohol or other drug use was reported. Of the 490 participants, 116 (23.7%) reported using alcohol or drugs in the prior 30 days at baseline, with a decline to 23 (4.7%) 6 months later (*p* < 0.001).

AIAN, American Indian and Alaska Native; AOD, alcohol and other drugs; CI, confidence interval; I/Ps, inmate/patients; TCP, traditional ceremonial practices.

### TCP measures

The studies included an array of TCP interventions or activities. The most common were drumming (*n* = 9), sweat lodge (*n* = 7), and talking circles (*n* = 6). All the studies included interventions that used TCPs that were either overseen or directly delivered by a traditional practitioner. The structure of the TCP measures varied broadly from a rigorous delivery schedule to cross-sectional surveys asking about participation in TCP during a specific period (e.g., last 12 months).

Several studies also included information about how TCP delivery was tailored depending upon tribal homogeneity, or lack thereof, with more heterogeneous communities choosing to adapt TCP based upon wide-ranging regional practices (e.g., the southwest or northern plains). For example, in their 2014 study, Dickerson et al.^31^ talk about how the processes of making drums and learning songs were adapted to meet the needs of a multi-tribal, urban community. This included drawing on traditional practitioners from different tribes as part of their cultural advisory board.

### Substance use behaviors

Each of the 10 studies reported some level of favorable change in substance use behaviors. The single RCT study^32^ found that participants who completed the TCP intervention reported fewer drinks per day (*d* = −0.39; confidence interval [95% CI] = −1.04 to 0.27) and lower odds of past 30-day marijuana use (odds ratio [OR] = 0.50; 95% CI = 0.10–2.54) compared to those participants who received similar hours of standard care. Five studies^31–34,35^ found TCP participation or interventions to be associated with a lower proportion of self-reported substance use behaviors. Three studies^28,36,37^ found that TCP participation was associated with either fewer days consuming alcohol or fewer alcoholic drinks consumed. One study^33^ reported lower substance use odds associated with TCP participation. And one study^38^ found that alcohol cessation was positively associated with participation in traditional spiritual activities.

### Quality of the included studies

In addition to cultural adherence, each study was evaluated for methodological quality using the RoB 2, ROBINS-I, or NIH checklist. The single RCT^32^ was ranked as having some concerns. Specifically, the researchers and staff involved in the study knew which participants were receiving the intervention during the trial, and it was not clear from the narrative whether the participants also knew. There was no indication of a high RoB, however, because the participants were randomized using a computer-generated randomization schedule, fidelity to the intervention was addressed throughout the study, and missing data were identified and accounted for in analysis.

Four of the cohort/evaluation studies^34,35,37,38^ were assessed as having a serious RoB and two^28,31^ as having a moderate RoB. Two of the cross-sectional studies^33,36^ scored 8 out of 14 and one^39^ scored 9 out of 14. While none of the studies was deemed as having a low RoB, there were also none considered to have a critical risk.

## Discussion

This review found suggestive evidence from 10 studies that TCP-based interventions can be protective against problem substance use among adult AIAN populations. Through experimental and observational designs, the included studies measured reductions in alcohol or other substance use associated with interventions and activities that are culturally congruent with AIAN traditional values, norms, and practices.

The need for TCPs as an intervention strategy is critical, as AIAN people continue to experience an epidemic of problem substance use. Yet, access to TCP-based interventions for AIAN communities is currently constrained by the longstanding gap between Indigenous practice-based evidence and Western science. This could be fueled by a lack of knowledge or even direct biases toward cross-cultural forms of problem substance use treatment. To redress these problems requires the promotion of science that incorporates responsible, quantitative research of Indigenous healing methods. TCPs are a culturally congruent strategy to reduce substance abuse. To our knowledge, this is the first systematic review to highlight quantitative data exploring the relationship between TCPs and problem substance use among AIAN adults.

Most substance abuse research examines the deficits, risks, and challenges within AIAN communities.^40–42^ This research currently fuels health policy and, subsequently, funding. Yet, identifying and quantifying the problem have not, in and of itself, provided a solution. Providing access to TCPs requires that clinicians and stakeholders be able to fund traditional forms of healing. However, this funding is too often hindered by either ignorance or bias against Indigenous research, despite millennia of practice and, perhaps the most confirmatory evidence, the continued existence and flourishing of AIAN communities in the face of significant traumas. This review focused on works that explore the inherent strengths and assets that AIAN communities possess, which create this environment for individuals to demonstrate resilience, a concept termed as *Reziliency*.^43^

While each of the 10 studies did include findings showing that TCPs and TCP-based interventions may be protective against problem substance use, it is important to note that there were also some unclear findings. The Dickerson et al.'s^31^ RCT study, for example, found lower rates of drinking and past 30-day marijuana use among the TCP-based treatment participants, but this trend reverses by the 3-month follow-up. They address this unexpected result in their discussion by outlining how their cultural advisory board members noted that once the intervention ended, it likely meant the participants no longer had the protection of TCP participation.

While more research is needed, this might highlight a potential danger of providing, then eliminating, access to TCPs upon completion of an intervention. Also, most of the studies did not break down substance use results by characteristics such as gender or age. So, while an overall decrease in substance use is reported, it cannot be known if this effect holds true for the entire population or only certain segments, for example, females or older adults.

### Reservation versus urban

One aim of this review was to identify whether there were differences in the application and/or effectiveness of TCP between reservation-based and urban AIAN communities. A noteworthy finding within the results is that the protective role of TCP does not appear to be confined to reservation societies. Studies in urban cities with tribally diverse samples reported reductions in problem substance use, which do not appear to differ significantly from those within tribally homogeneous communities.

This is especially meaningful, given that the majority of AIAN individuals no longer live on tribally controlled lands, but rather in urban settings where substance use programming would need to serve a multi-tribal population.^44^ For example, in their 2021 study, Dickerson et al.^32^ report that their urban sample included affiliations from 33 different tribes. Yet the program was still able to demonstrate a reduction in both number of drinks per day and marijuana use through its TCP-based intervention.

### Cultural integrity

As they gain in popularity, it is essential that TCPs continue to be overseen in design or directly led by an Indigenous traditional practitioner, as reflected in each of the studies included in this review. It is critical that these current results be interpreted to reflect not just on the individual activities, such as drumming, smudging, or sweating, which traditional wisdom dictates are not independent, severable components. Rather, researchers must look on these as collective practices that are contextual and interwoven with language, geography, and tribal histories. The context of this evidence dictates that programming not be undertaken irreverent of the spiritual and traditional element, or, as one qualitative study title directly calls out, “Please Don't Just Hang a Feather on a Program or Put a Medicine Wheel on Your Logo and Think ‘Oh Well, This Will Work…”^45^

It is also important to note that many studies examining TCP do not provide definitions or explanations as to how TCP was operationalized or carried out. This was a significant hurdle for this review, as there were likely several studies—such as the Venner et al.'s studies^29,30^ noted earlier—that could not be included because it was not possible from the article narrative to parse out or even determine the use of TCP. If more specific information were available, these same studies might have been included.

While inclusion of those studies might have amplified these results, there are very sincere and serious reasons why many AIAN communities may not be comfortable sharing the level of detail about their practices that would allow for inclusion. It would be naive and irresponsible for any researcher to expect tribal communities to ignore past events—including the historical criminalization of the very ceremonies being practiced now and the imprisonment or murder of practitioners leading those ceremonies—when reporting on studies today.

### Practical implications

It is the hope of this research team that to the greatest extent possible, this review will support and inform the development and delivery of substance use programming for AIAN communities. In addition to specific activities and practices, several common themes were identified and laid out from studies that incorporate TCP aiming to reduce problem substance use: (1) the process of designing and evaluating programming and research should either be led or informed by AIAN communities; (2) TCPs should be delivered or overseen by a legitimate traditional practitioner; and (3) TCPs are not severable components and should be delivered within the context of spiritual and traditional elements.

To assist with future evaluation of the effectiveness of TCP, up to and including meta-analyses, researchers could adopt some standardized measures, while still allowing the adaptation of programming to reflect tribal affiliations among the community. Specifically, some feasible measures to include in future studies appear to be ORs for substance use versus abstinence, cross-tabulations (chi-square) between TCP and non-TCP subsamples, number of days using a particular substance in the last 30 days, and the quantity of substance consumed per day, as appropriate.

### Declaration of Astana—living out a commitment to health

A key component of the 2018 *Declaration of Astana* is the acknowledgment of premature loss of life due to noncommunicable diseases, such as addiction, and the need to promote opportunities for improved health, especially among marginalized and underserved communities.^6^ While this review has several limitations that are outlined below, there is at least suggestive evidence that TCP-based interventions may be effective, sustainable approaches to reducing problem substance use and mortality in AIAN communities throughout the United States.

The *Declaration of Astana* also acknowledges the need to draw on traditional knowledge and wisdoms in developing policies and practices to address health disparities.^6^ Not only are TCP-based practices derived from millennia of traditional knowledge but they are also community driven and community centered. While a majority of public and private funders have chosen to establish clinically derived evidence-based criteria when selecting programs and services they will support, this does not prevent them from making concurrent investments in culturally centered, community-led initiatives such as those found in this review.

### Strengths and limitations

A significant strength of this review was the strong Native American representation and extensive clinical and research experience with AIAN people in the authorship. This review was led by a Native American author with more than two decades of professional experience in AIAN health and whose lived experience includes practicing and participating in TCP. The senior member of the research team is a Native American clinical psychologist whose research has focused on identifying and enhancing culturally based protective factors. The two non-Native researchers have a background working with tribal and urban AIAN communities and one previously led a similar systematic review focused on TCP and preventing substance abuse among AIAN youth.

An immediate limitation is the rarity of studies in general, which report quantitative data regarding TCP. Several systematic or scoping reviews have highlighted qualitative evidence that supports the effectiveness and promise of TCP to reduce problem substance use. While qualitative methodologies allow for a much deeper and contextualized understanding of phenomena, evaluation of the effectiveness of TCP-based interventions must necessarily include quantitative measures as well. Currently, this is largely missing in the published literature. Because of this, it was not possible to complete a meta-analysis due to the lack of consistent statistical methods among the few studies that did publish this type of data. In addition, the scarcity of studies did not enable us to compare the effect based on type of substance use (i.e., alcohol, marijuana, or other drugs). It is possible that more data could be found in future searches of gray literature available on this topic.

The OneSearch function of the library search platform unfortunately did not delineate results based on which particular database(s) returned which articles. Instead, the full list of references returned from the search strategy (*n* = 5669) were uploaded into EndNote, plus an additional 18 that were not in the original search, but were found by reviewing the references of full text articles. These references were screened for duplicates and the remaining articles (*n* = 4022) were stored in a master folder on EndNote and are available upon request.

A further limitation was the subjective process used to review for cultural bias. The criteria applied were established by this review team after lengthy discussion. They are not based on a widely applied, standardized approach to study evaluation. While the authors strived in earnest to apply the criteria as rigorously and defensibly as possible, further work in this area is needed to flush out a more uniform process in future reviews.

Finally, the belief of this review team that TCPs should be defined by respective communities as both a strength and a limitation. While in line with the tenets of CIRM, it limits generalizability. For example, a TCP-based intervention designed and delivered in the Pacific Northwest might not show the same effectiveness, without significant adaptation, if employed in an AIAN community in the Southeast. However, this reinforces the finding and recommendation that TCP-based interventions be community led and either overseen or delivered by legitimate traditional practitioners.

## Conclusions

AIAN communities have relied on the strength of TCPs to preserve and promote wellness since time immemorial. While the drive to pour funding into substance use programming and services with a strong evidence base is a well-intentioned approach to responsible stewardship of limited resources, it is, nevertheless, a myopic approach. The lack of translation from practice-based evidence to clinical data regarding the effectiveness of TCPs stems from very serious barriers: the historical criminalization of TCPs; recurrent disregarding of TCPs as baseless superstition by Western society; and a resulting, persistent distrust of research on the part of AIAN communities.

While there was not enough synthesizable quantitative data to perform a meta-analysis as part of this review, the existing literature does indicate promise for the use of TCPs to address problem substance use in AIAN communities in a way that is effective and also culturally congruent. This review intended to elucidate the potential benefits of TCP-based substance use interventions, with the hope that emerging and future research will help establish a more robust evidence base. Given the evidence, the review team is cautiously encouraged and believes this topic merits further research and attention.

## Supplementary Material

Supplemental data

Supplemental data

## Supplementary Material

Supplementary Appendix SA1

Supplementary Appendix SA2

## Notes

^*^
The authors subscribe to the belief that the most appropriate way to refer to any Indigenous person or tribe is using the term from their specific language or dialect. For consistency, throughout this article, we will broadly refer to Indigenous persons and peoples of the United States using the widely accepted term American Indian and Alaska Native (AIAN). However, other terms such as Indigenous, Native American, Native, American Indian, or Indian may be substituted based on context or original study language.

^*^
**Correction added:** On May 26, 2023 after first online publication of April 8, 2023: The first/lead author has had a legal name change from D'Shane Barnett to Damian M. Chase-Begay. The byline has been amended to reflect the change.

^*^
**Correction added:** On May 26, 2023 after first online publication of April 8, 2023: The author has had a legal name change from D'Shane Barnett to Damian M. Chase-Begay. Additionally the email address has changed from dshane.barnett@umt.edu to damian.chasebegay@umt.edu
