# Supplementary material for: Traditional Ceremonial Practices as a Strategy to Reduce Problem Substance Use in American Indian Communities: A Systematic Review
Source: J Integr Complement Med. 2023 Jun 6;29(6-7):408–19. doi: 10.1089/jicm.2022.0655 (PMC10280199; doi:10.1089/jicm.2022.0655)
Supplement: Supplemental data [file Suppl_AppendixSA1.pdf]

SUPPLEMENTAL APPENDIX SA1. Systematic Review Protocol

National Institute for Health Research

UNIVERSITY of York
Centre for Reviews and Dissemination

Systematic review

1. * Review title.

Give the title of the review in English

Ceremony and traditional spiritual practices (CTSP) as a culturally tailored strategy against problem substance use in urban American Indian communities: A systematic review

2. Original language title.

For reviews in languages other than English, give the title in the original language. This will be displayed with the English language title.

3. * Anticipated or actual start date.

Give the date the systematic review started or is expected to start.

01/09/2021

4. * Anticipated completion date.

Give the date by which the review is expected to be completed.

31/12/2021

5. * Stage of review at time of this submission.

Tick the boxes to show which review tasks have been started and which have been completed. Update this field each time any amendments are made to a published record.

Reviews that have started data extraction (at the time of initial submission) are not eligible for inclusion in PROSPERO. If there is later evidence that incorrect status and/or completion date has been supplied, the published PROSPERO record will be marked as retracted.

This field uses answers to initial screening questions. It cannot be edited until after registration.

The review has not yet started: Yes

Review stage	Started	Completed
Preliminary searches	No	No
Piloting of the study selection process	No	No
Formal screening of search results against eligibility criteria	No	No
Data extraction	No	No
Risk of bias (quality) assessment	No	No
Data analysis	No	No

Provide any other relevant information about the stage of the review here.

6. * Named contact.

The named contact is the guarantor for the accuracy of the information in the register record. This may be any member of the review team.

D'Shane Barnett

Email salutation (e.g. "Dr Smith" or "Joanne") for correspondence:

D'Shane

7. * Named contact email.

Give the electronic email address of the named contact.

dshane.barnett@umontana.edu

8. Named contact address

Give the full institutional/organisational postal address for the named contact.

32 Campus Drive

Skaggs 177

9. Named contact phone number.

Give the telephone number for the named contact, including international dialling code.

4063049494

10. * Organisational affiliation of the review.

Full title of the organisational affiliations for this review and website address if available. This field may be completed as 'None' if the review is not affiliated to any organisation.

University of Montana

Organisation web address:

https://health.umt.edu/publichealth/

11. * Review team members and their organisational affiliations.

Give the personal details and the organisational affiliations of each member of the review team. Affiliation refers to groups or organisations to which review team members belong. **NOTE: email and country now MUST be entered for each person, unless you are amending a published record.**

Mr D'Shane Barnett. University of Montana
Jeffery Peterson. University of Montana
Jessica Liddell. University of Montana
Annie Belcourt. University of Montana

12. * Funding sources/sponsors.

Details of the individuals, organizations, groups, companies or other legal entities who have funded or sponsored the review.

None

Grant number(s)

State the funder, grant or award number and the date of award

13. * Conflicts of interest.

List actual or perceived conflicts of interest (financial or academic).

None

14. Collaborators.

Give the name and affiliation of any individuals or organisations who are working on the review but who are not listed as review team members. **NOTE: email and country must be completed for each person, unless you are amending a published record.**

15. * Review question.

State the review question(s) clearly and precisely. It may be appropriate to break very broad questions down into a series of related more specific questions. Questions may be framed or refined using PI(E)COS or similar where relevant.

Is there an association between prevention or treatment interventions that incorporate ceremony and traditional spiritual practices (CTSP) for American Indians who live on tribal lands and problem substance use?

Is there an association between prevention or treatment interventions that incorporate ceremony and traditional spiritual practices (CTSP) for American Indians who live in non-tribal, urban settings and problem substance use?

16. * Searches.

State the sources that will be searched (e.g. Medline). Give the search dates, and any restrictions (e.g. language or publication date). Do NOT enter the full search strategy (it may be provided as a link or attachment below.)

The following databases will be searched: (1) PubMed, (2) Global Health, (3) Global Health Archive, (4)

CINAHL, (5) PsycINFO, (6) Web of Science, (7) Health and Wellness (Gale), (8) SAGE, (9) ScienceDirect, and (10) Google Scholar. Studies are eligible for review if they are published in English and the full text is available.

17. URL to search strategy.

Upload a file with your search strategy, or an example of a search strategy for a specific database, (including the keywords) in pdf or word format. In doing so you are consenting to the file being made publicly accessible. Or provide a URL or link to the strategy. Do NOT provide links to your search **results**.

https://www.crd.york.ac.uk/PROSPEROFILES/269710_STRATEGY_20210828.pdf

Alternatively, upload your search strategy to CRD in pdf format. Please note that by doing so you are consenting to the file being made publicly accessible.

Do not make this file publicly available until the review is complete

18. * Condition or domain being studied.

Give a short description of the disease, condition or healthcare domain being studied in your systematic review.

The outcome of interest in this review is substance use disorder (SUD), including alcohol, methamphetamine, and opioids.

19. * Participants/population.

Specify the participants or populations being studied in the review. The preferred format includes details of both inclusion and exclusion criteria.

The population of interest is Indigenous (American Indian/Native American) adults (age 18 and over) within the United States, including Alaska and Hawaii.

20. * Intervention(s), exposure(s).

Give full and clear descriptions or definitions of the interventions or the exposures to be reviewed. The preferred format includes details of both inclusion and exclusion criteria.

Interventions aimed at preventing, treating, or reducing substance use in American Indian adults that incorporate ceremony or other traditional spiritual practices.

21. * Comparator(s)/control.

Where relevant, give details of the alternatives against which the intervention/exposure will be compared (e.g. another intervention or a non-exposed control group). The preferred format includes details of both inclusion and exclusion criteria.

No intervention or interventions aimed at preventing, treating, or reducing substance use in American Indian adults that do not incorporate ceremony or other traditional spiritual practices.

22. * Types of study to be included.

Give details of the study designs (e.g. RCT) that are eligible for inclusion in the review. The preferred format includes both inclusion and exclusion criteria. If there are no restrictions on the types of study, this should be stated.

Included in the review: Quantitative studies, including clinical trials, comparative studies, evaluation studies, and observational studies. Excluded from the review: Qualitative studies.

23. Context.

Give summary details of the setting or other relevant characteristics, which help define the inclusion or exclusion criteria.

Prevention programming, treatment services, or community-based practices that report on substance use outcomes for Indigenous participants.

24. * Main outcome(s).

Give the pre-specified main (most important) outcomes of the review, including details of how the outcome is defined and measured and when these measurement are made, if these are part of the review inclusion criteria.

Impacts on substance use, including number of times or days when a substance is used, amount of substance(s) consumed, age of initiation of use, or changes in other objective scale measurements.

Measures of effect

Please specify the effect measure(s) for you main outcome(s) e.g. relative risks, odds ratios, risk difference, and/or 'number needed to treat.

25. * Additional outcome(s).

List the pre-specified additional outcomes of the review, with a similar level of detail to that required for main outcomes. Where there are no additional outcomes please state 'None' or 'Not applicable' as appropriate to the review

Is there a difference in effect between American Indians who live on tribal lands vs those who live in non-tribal, urban settings.

Measures of effect

Please specify the effect measure(s) for you additional outcome(s) e.g. relative risks, odds ratios, risk difference, and/or 'number needed to treat.

26. * Data extraction (selection and coding).

Describe how studies will be selected for inclusion. State what data will be extracted or obtained. State how this will be done and recorded.

EndNote 20 will be used to manage all references for this review. The databases will be searched independently by two of the reviewers (DSB, JP) using the established search strategy. Initial citations will be combined and duplicates will be removed. DSB and JP will then independently screen all citations first by title, then by abstract, for inclusion in the review. For any citation without an abstract, full text will be retrieved and reviewed by DSB and JP. Any citation not eliminated by both reviewers based on title or abstract will move to full text review.

Full text review will take place by DSB and JP. A fillable form will be used by both reviewers to track the following data: (a) year of study; (b) study design; (c) study population; (d) intervention/exposure measures; and (e) outcome measures.

Any disagreement on inclusion during the full text stage of review will be reconciled by the full review team through consensus or lead author final say, if necessary. A PRISMA flow chart will be used to document exclusion throughout the process.

27. * Risk of bias (quality) assessment.

State which characteristics of the studies will be assessed and/or any formal risk of bias/quality assessment tools that will be used.

Cohort studies, quasi-randomized trials, case-control studies, cross-sectional studies, interrupted time series and controlled before-after studies will all be assessed using the Risk Of Bias In Non-randomized Studies of Interventions (ROBINS-I) [1]. Any randomized control trials will be assessed using the Revised Cochrane risk-of-bias tool for randomized trials (RoB 2) [2]. Any observational studies that cannot be assessed using ROBINS-I or RoB 2 will be assessed using the STrengthening the Reporting of OBservational studies in Epidemiology (STROBE) checklist [3].

In addition to Western quality assessment protocols, Indigenous members of the research team will provide a review of cultural competency within studies as it relates to potential risk of bias.

[1] Sterne JAC, Hernán MA, Reeves BC, et al. ROBINS-I: a tool for assessing risk of bias in non-randomized studies of interventions. BMJ 2016; 355; i4919.
[2] Revised Cochrane risk-of-bias tool for randomized trials (RoB 2). Edited by Julian PT Higgins, Jelena Savovi?, Matthew J Page, Jonathan AC Sterne on behalf of the RoB2 Development Group. 22 August 2019.
[3] Cuschieri S. The STROBE guidelines. Saudi J Anaesth. 2019 Apr;13(Suppl 1):S31-S34. doi: 10.4103/sja.SJA_543_18. PMID: 30930717; PMCID: PMC6398292.

28. * Strategy for data synthesis.

Describe the methods you plan to use to synthesise data. This **must not be generic text** but should be **specific to your review** and describe how the proposed approach will be applied to your data. If meta-analysis is planned, describe the models to be used, methods to explore statistical heterogeneity, and software package to be used.

Quantitative data synthesis: Our greatest hope is to find quantitative data that can be synthesized via a meta-analysis. We expect many, if not most, of the studies to include cross-sectional design. Using the Review Management (RevMan) software, we will calculate pooled odds ratios for those studies with sufficient similarity. Clinical heterogeneity will be assessed by two authors (DSB, JP) and statistical heterogeneity will be assessed via the I^2 statistic. Studies will be individually excluded from analysis to determine their impact on the results. A funnel plot will be used to assess potential publication bias.

Qualitative data analysis: For those studies that cannot be meta-analyzed for overall effect due to excessive heterogeneity, we will use NVivo software to employ a qualitative descriptive analysis [1]. Themes and

subthemes will be identified and coded, and quotes or other relevant text will be used to report on findings.

[1] Colorafi KJ, Evans B. Qualitative Descriptive Methods in Health Science

Research.HERD.2016;9(4):16-25.

29. * Analysis of subgroups or subsets.

State any planned investigation of ‘subgroups’. Be clear and specific about which type of study or participant will be included in each group or covariate investigated. State the planned analytic approach.

Depending on the available data, the following subgroups might be investigated:

-Participants on tribally-controlled lands vs non-tribal, urban settings

-Type of substance use: alcohol, marijuana, methamphetamine, inhalant, opioid

-Multiple substance user vs single substance user

-Higher enculturation vs higher acculturation

30. * Type and method of review.

Select the type of review, review method and health area from the lists below.

Type of review

Cost effectiveness
No

Diagnostic
No

Epidemiologic
No

Individual patient data (IPD) meta-analysis
No

Intervention
No

Living systematic review
No

Meta-analysis
Yes

Methodology
No

Narrative synthesis
Yes

Network meta-analysis
No

Pre-clinical
No

Prevention
No

Prognostic
No

Prospective meta-analysis (PMA)
No

Review of reviews
No

Service delivery
No

Synthesis of qualitative studies
No

Systematic review
Yes

Other
No

Health area of the review

Alcohol/substance misuse/abuse
Yes

Blood and immune system
No

Cancer
No

Cardiovascular
No

Care of the elderly
No

Child health
No

Complementary therapies
No

COVID-19
No

Crime and justice
No

Dental
No

Digestive system
No

Ear, nose and throat
No

Education
No

Endocrine and metabolic disorders

No

Eye disorders
No

General interest
No

Genetics
No

Health inequalities/health equity
No

Infections and infestations
No

International development
No

Mental health and behavioural conditions
No

Musculoskeletal
No

Neurological
No

Nursing
No

Obstetrics and gynaecology
No

Oral health
No

Palliative care
No

Perioperative care
No

Physiotherapy
No

Pregnancy and childbirth
No

Public health (including social determinants of health)
No

Rehabilitation
No

Respiratory disorders
No

Service delivery
No

Skin disorders
No

Social care
No

Surgery
No

Tropical Medicine
No

Urological
No

Wounds, injuries and accidents
No

Violence and abuse
No

31. Language.

Select each language individually to add it to the list below, use the bin icon to remove any added in error.
English

There is not an English language summary

32. * Country.

Select the country in which the review is being carried out. For multi-national collaborations select all the countries involved.

United States of America

33. Other registration details.

Name any other organisation where the systematic review title or protocol is registered (e.g. Campbell, or The Joanna Briggs Institute) together with any unique identification number assigned by them. If extracted data will be stored and made available through a repository such as the Systematic Review Data Repository (SRDR), details and a link should be included here. If none, leave blank.

34. Reference and/or URL for published protocol.

If the protocol for this review is published provide details (authors, title and journal details, preferably in Vancouver format)

Add web link to the published protocol.

Or, upload your published protocol here in pdf format. Note that the upload will be publicly accessible.

No I do not make this file publicly available until the review is complete

Please note that the information required in the PROSPERO registration form must be completed in full even if access to a protocol is given.

35. Dissemination plans.

Do you intend to publish the review on completion?

Yes

Give brief details of plans for communicating review findings.?

We expect to publish in a peer-related journal within the public health, substance use, American Indian health, or other applicable field.

36. Keywords.

Give words or phrases that best describe the review. Separate keywords with a semicolon or new line. Keywords help PROSPERO users find your review (keywords do not appear in the public record but are included in searches). Be as specific and precise as possible. Avoid acronyms and abbreviations unless these are in wide use.

American Indian
Native American

Substance Use

Ceremony

Traditional Practices

37. Details of any existing review of the same topic by the same authors.

If you are registering an update of an existing review give details of the earlier versions and include a full bibliographic reference, if available.

38. * Current review status.

Update review status when the review is completed and when it is published.New registrations must be ongoing so this field is not editable for initial submission.
Please provide anticipated publication date

Review_Ongoing

39. Any additional information.

Provide any other information relevant to the registration of this review.

40. Details of final report/publication(s) or preprints if available.

Leave empty until publication details are available OR you have a link to a preprint (NOTE: this field is not editable for initial submission). List authors, title and journal details preferably in Vancouver format.

Give the link to the published review or preprint.
