# Supplementary material for: Traditional Ceremonial Practices as a Strategy to Reduce Problem Substance Use in American Indian Communities: A Systematic Review
Source: J Integr Complement Med. 2023 Jun 6;29(6-7):408–19. doi: 10.1089/jicm.2022.0655 (PMC10280199; doi:10.1089/jicm.2022.0655)
Supplement: Supplemental data [file Suppl_AppendixSA2.pdf]

SUPPLEMENTAL APPENDIX SA2. Abstract and Article Screening Guidelines

Citation, Title, and Abstract Screening

1. Does the **title or abstract** use English?
    a. Yes: continue screening
    b. No: stop screening

2. Does the **title or abstract** NOT indicate that a TCP systematic review or meta-analysis was conducted?
    a. Yes: continue screening
    b. No: stop screening

3. Does the **title or abstract** indicate that this is NOT a correction or erratum?
    a. Yes: continue screening
    b. No: stop screening

Abstract Screening

4. Does the **abstract** indicate that an AIAN adult sample was studied?
    a. Yes or Unsure/Unclear: continue screening
    b. No: stop screening
        -For example: the study only samples youth under age 18 or adults who are not AIAN

5. Does the **abstract** indicate that this study was quantitative?
    a. Yes or Unsure/Unclear: continue screening
        -Key words: Quantitative, clinical trial, randomize, pilot study, comparative, evaluation, and observational
    b. No: stop screening
        -For example: the study only used qualitative methods
        -Key point: some studies may employ both qualitative and quantitative methods and these should be kept in the screening process

6. Does the **abstract** indicate that AIAN TCP was studied?
    a. Yes or Unsure/Unclear: continue screening
        -Key words: ceremony, traditional practice(s), cultural practice(s), spirituality, ritual(s), rite(s), American Indian, Native American, Indigenous, and tribal practice(s)
    b. No: stop screening
        -Other constructs, in the absence of TCP measures above, **not** eligible: Western religious practices or evidence-based and best practices that do not include a measurable TCP component (e.g., AA, cognitive behavioral therapy, medication-assisted treatment)

7. Does the **abstract** indicate that substance use prevention or treatment was studied?
    a. Yes: continue screening

-Key words: alcohol, marijuana, methamphetamines, opioids, heroin, cocaine, inhalants, drug(s), illicit substance(s), substance (ab)use, alcoholism, and abstinence
-Key point: If the study clearly mentions any type of substance use as either a direct or indirect measure, then assume substance use was studied

b. No: stop screening
-Key point: mental illness, physical health conditions, and tobacco use (smoking, vaping, and smokeless products) are NOT eligible

Decision: **Should full text of the article be screened?**

a. **Yes**, all 7 screening questions answered either "Yes" or "Unclear"
b. **No**, at least one screening question answer is definitely "No"

Full Article Review (all answers must be "Yes" to be included in the review)

8. Does the **article** provide information about an AIAN adult sample?
9. Does the **article** include at least one quantitative measure regarding TCP interventions or activities?
10. Does the **article** include at least one quantitative measure regarding use of eligible substances?
11. Does the **article** indicate that the research study was either Indigenous-led or Indigenous-informed?
12. Does the **article** indicate that TCP interventions or activities were provided or overseen by a traditional practitioner?
13. Does the **article** provide a justification that the implementation and/or evaluation procedures were culturally appropriate for the target population?

Decision: **Should the article be included in the review?**

a. **Yes**, questions 8-13 answered with all "Yes"
b. **No**, at least one answer to questions 8-13 is definitely "No"
c. **Potentially,** questions 8-13 answered with at least one "Unclear" but without any definite "No" – bring to full team for discussion and consensus
